# The DASSL or Data Access, Storage, Sharing and Linkage Model & Proposals for an Enabling Data Environment for Health and Related Research in Ireland

**DOI:** 10.23889/ijpds.v1i1.303

**Published:** 2017-04-19

**Authors:** Rosalyn Moran

**Affiliations:** Health Research Board

## Objectives

This project set out 1)to examine the barriers and facilitators which impact on researchers' ability to access, share and link data and thus carry out health and related research in Ireland and 2)to explore possible solutions.

## Approach

Interviews were conducted with individuals involved in the collection, use, storage, sharing and linkage of data in Ireland. Data-related needs, practices and attitudes were explored, as were respondents' views regarding both existing and required infrastructure and services for the safe sharing and linkage of data. Ongoing discussions with key stakeholders, visits to centres of excellence abroad and a review of the international literature informed results.

## Results

Based on the work carried out a model for safe access, sharing and linkage of data within the Irish context was developed - the DASSL or data access, storage, sharing and linkage model. The model comprises seven elements - five related to infrastructure and services [a health research data hub, safe haven, trusted third party and data linkage service, output checking and disclosure control and a research support unit] required for safeguarding data, and two related to the broad legislative and socio-cultural context needed to facilitate implementation of the model i.e. governance and public engagement. It is suggested that a research data trust [RDT] would be established to house the DASSL elements and to provide the institutional and technical environment to respond to the growing data-related needs within the Irish health research environment and the broader data ecosystem.

## Conclusion

The proposals put forward for creating a safe environment for access, sharing and linkage of research and related data need to be considered by all key stakeholders. It is suggested that a Data to Benefits Committee be established to drive strategic discussions and action towards the implementation of the DASSL model elements. Implementation of the DASSL model will allow for safe usage of currently under-exploited data which can help inform health and wellbeing but also serve national economic and social agendas.

